# A Novel Multi-Exposure Image Fusion Method Based on Adaptive Patch Structure

**DOI:** 10.3390/e20120935

**Published:** 2018-12-06

**Authors:** Yuanyuan Li, Yanjing Sun, Mingyao Zheng, Xinghua Huang, Guanqiu Qi, Hexu Hu, Zhiqin Zhu

**Affiliations:** 1 School of Information and Electrical, China University of Mining and Technology, Xuzhou 221116, China; 2 College of Automation, Chongqing University of Posts and Telecommunications, Chongqing 400065, China; 3 College of Automation, Chongqing University, Chongqing 400044, China; 4 Department of Mathematics and Computer Information Science, Mansfield University of Pennsylvania, Mansfield, PA 16933, USA; 5 School of Computing, Informatics, and Decision Systems Engineering, Arizona State University, Tempe, AZ 85287, USA

**Keywords:** multi-exposure image fusion, texture information entropy, adaptive selection, patch structure decomposition

## Abstract

Multi-exposure image fusion methods are often applied to the fusion of low-dynamic images that are taken from the same scene at different exposure levels. The fused images not only contain more color and detailed information, but also demonstrate the same real visual effects as the observation by the human eye. This paper proposes a novel multi-exposure image fusion (MEF) method based on adaptive patch structure. The proposed algorithm combines image cartoon-texture decomposition, image patch structure decomposition, and the structural similarity index to improve the local contrast of the image. Moreover, the proposed method can capture more detailed information of source images and produce more vivid high-dynamic-range (HDR) images. Specifically, image texture entropy values are used to evaluate image local information for adaptive selection of image patch size. The intermediate fused image is obtained by the proposed structure patch decomposition algorithm. Finally, the intermediate fused image is optimized by using the structural similarity index to obtain the final fused HDR image. The results of comparative experiments show that the proposed method can obtain high-quality HDR images with better visual effects and more detailed information.

## 1. Introduction

**Figure 1:**
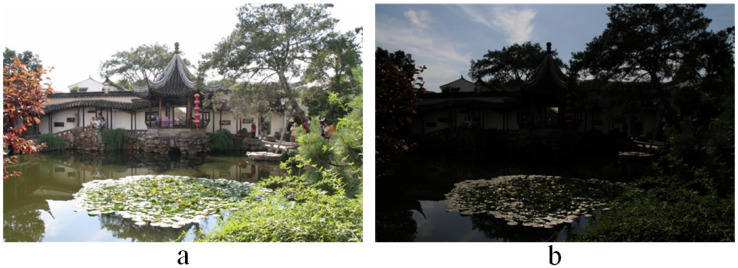
(**a**) Over-exposure image; (**b**) under-exposure image.

Due to the limited dynamic range of imaging devices, it is not possible to capture all the details in one scene by a single exposure with existing imaging devices [1,2]. This seriously affects image visualization and the demonstration of key information. Figure 1a shows an over-exposed image. When shooting requires a long exposure time, the imaging device can effectively capture the information from the dark part. However, due to the over-exposure, the details of the bright part get severely lost. On the contrary, when the exposure time is short, the information of the bright part is captured, but the information of the dark part is lost. As shown in Figure 1b, the under-exposure phenomenon is caused by the mismatch of the dynamic range between the human visual system and electronic imaging devices [1].

Multi-exposure image fusion (MEF) methods provide an effective way to solve the mismatch of dynamic range among existing imaging devices, display equipment, and human eyes’ response to real scenes. It takes source image sequences of different exposure levels as the inputs. An informative and perceptive high-dynamic-range (HDR) image is generated by synthesizing the information of the luminance conforming to the human visual system [3]. The fused image contains more abundant scene luminance, color, and detailed information, which make the image correspond to the real scene observed by the human eye [4,5]. In addition, it also provides more information for subsequent image processing [6]. MEF algorithms are mainly categorized as transform domain- and spatial domain-based fusion algorithms.

Transform domain-based fusion methods have three main steps: First, source image sequences are decomposed into the transform domain. Then, fusion coefficients from the source images are selected according to the fusion rules. Finally, the fused image is obtained by inversely transforming the fusion coefficients [7,8,9]. Based on the Laplacian pyramid, Mertens proposed multiple resolutions to fuse an exposure sequence into an HDR image [10]. The weighted value determined by contrast, saturation, and well-exposedness took a weighted average to obtain the pyramid coefficients. The obtained pyramid coefficients were reconstructed to get the final image. Li performed two-scale decomposition on source images to obtain the image base layer and the detail layer first. Then, the spatial consistency was applied to fuse the obtained base and detail layers to get the fused image [11]. Bruce introduced nonlinearity to balance the visible details and smoothness of the fused result to capture the information present in the source images [12]. Kou applied the weighted-least-squares-based image smoothing algorithm to one MEF algorithm for detail extraction in an HDR scene [13]. The extracted details were then used in the multi-scale exposure fusion algorithm to achieve image fusion. Based on the hybrid exposure weight and the novel boosting Laplacian pyramid (BLP), an exposure fusion approach proposed by Shen considered gradient vectors among different exposure source images [14]. This method used the improved Laplacian pyramid to decompose input signals into base and detail layers. Then, a fused image with rich color and detailed information could be obtained. As a shortcoming of transform domain-based fusion algorithms, when the luminance range of the real scene is large, the useful details of the over-exposure and under-exposure regions are lost. This seriously affects the visual effects of the fused image.

Source images from different exposures can also be fused by spatial domain-based fusion methods [15,16,17]. Spatial domain fusion methods have two main types: image patch- and pixel-based fusion [5,18,19]. As a pixel-based fusion algorithm, Shen proposed a probabilistic model for MEF [20]. Subject to two quality measures, such as the local contrast and color consistency of source image sequence, the proposed model calculated an optimal set of probabilities by using a generalized random walk framework. The probability sets were then used as weights to achieve image fusion. Based on the probabilistic model [20,21], Shen proposed another MEF method by integrating the perceptual quality measure [22]. In this method, the probability of the human visual system was modeled by contrast and color information, as well as the optimal fusion weight obtained by using the hierarchical multivariate Gaussian conditional random field. This method can improve MEF performance and provide a better visual experience for audiences. Gu introduced a new method for MEF that obtained the gradient value of the pixel by maximizing the structure tensor [23]. The local gradient was used to represent pixel contrast first. Then, the fused image was obtained by the inverse transformation of the gradient field. Li established a multi-exposure image fusion model based on median filtering and recursive filtering [24]. It made a comprehensive evaluation of different regions of the multi-exposure image and fused those pixels from median filtering and recursive filtering over contrast, color, and brightness exposure. This method reduced the computational complexity of effective fusion. On the basis of the rank-one structure of low-dynamic-range (LDR) images, Tae-Hyun proposed a MEF image algorithm [6]. This algorithm formulated HDR generation as a rank minimization problem (RMP) that simultaneously estimated a set of geometric transformations to align LDR images and detected both moving objects and under-/over-exposed regions. Since pixel-based MEF algorithms involved averaging pixels to obtain fused pixels, this method reduced the sharpness and contrast, which affected the visual quality of fused image.

Owing to the image patches, Song proposed a fusion method to suppress gradient inversion by integrating the details of the local adaptive scene [25]. A variational method that combined color matching and gradient information was proposed by Bertalmio to achieve image fusion [26]. It used short- and long-exposure images to measure differences in edge information and local chromatic aberrations, respectively. Zhang used the contrast standard to measure the quality of details in the exposure and kept the details in intermediate images [27]. By combining intermediate images seamlessly, an HDR image with rich details could be generated. According to the principle of the structural similarity (SSIM) index [28], Ma proposed a structural patch decomposition-based MEF for image fusion [3]. This method can produce noise-free weighted mapping, more natural color information, and a high-quality fused image. Based on the decomposition of the image patch structure [3], Ma introduced a color structural similarity (SSIMc) index to achieve multi-exposure image fusion [29]. The source image spaces were explored in an iterative process by the gradient ascent algorithm to search for an image with optimized SSIMc. Finally, a high-quality fused image with a realistic structure and vivid color was obtained. Compared with pixel-based fusion methods, patch-based fusion methods do not average pixels, but can obtain a fused image with better sharpness and contrast. Since image patch size in patch-based fusion algorithms is fixed, it causes the fused image to lose fine-detail information of the structure and texture in the multi-exposure source image sequence.

In order to obtain high-quality HDR images, this paper proposes an MEF algorithm based on adaptive patch structure (APS-MEF), which retains more detailed information of the scene. First, input multi-exposure source images are subjected to cartoon-texture decomposition, and the adaptive decomposition of the image patch is realized by calculating the entropy of the image texture [30]. This is helpful to improve the robustness of image patch decomposition. Then, three components, the signal strength, signal structure, and mean intensity, are obtained by applying structural patch decomposition. The initial fused image is obtained by processing three components separately. The decomposition algorithm of the structural patch processes three color channels at the same time, which can capture more color information of the source scene and obtain a more vivid fused image. Finally, the SSIMc is applied to optimize the initial fused image to balance both local and overall brightness. The fused image is consistent with the human visual system. Compared with five MEF methods in 24 different scenes, the experiment results confirm that the proposed APS-MEF method can retain more detailed information and generate high-quality fused images. Based on the adaptive selection of the image patch size, the proposed fusion algorithm brings three main contributions to traditional MEF methods:

- It uses texture entropy to evaluate image information, which has strong adaptability and robustness.
- It implements the adaptive selection of image patch size by measuring texture entropy, which enables the fused image to retain more detailed information of the source images.
- It combines image cartoon-texture decomposition, image patch structure decomposition, and SSIM index optimization to adjust the local brightness and makes fused images sharper and more smooth.

The rest of this paper is organized as follows: Section 2 presents the APS-MEF algorithm in detail; Section 3 discusses comparative experiments; and Section 4 concludes this paper.

## 2. MEF Framework Based on the Adaptive Selection of Image Patch Size

**Figure 2:**
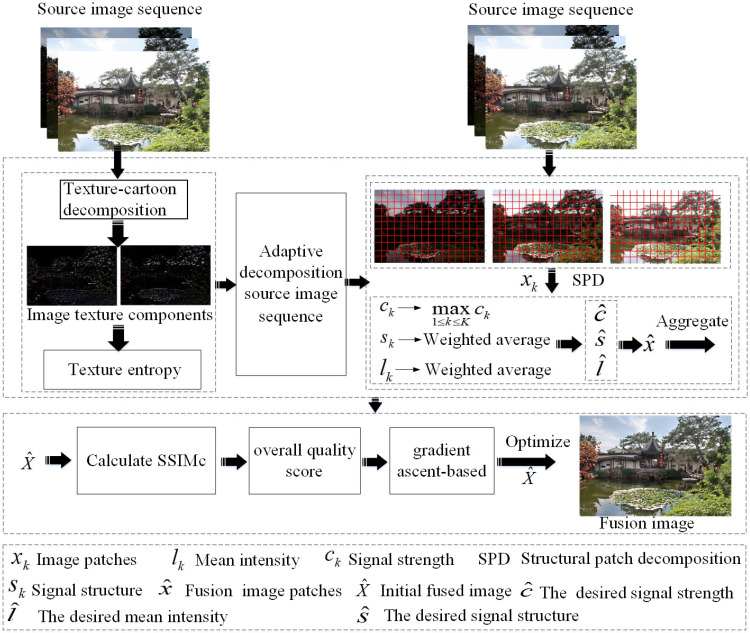
The proposed adaptive patch structure multi-exposure image fusion (APS-MEF) framework. SSIMc: color structural similarity.

Based on the adaptive selection of image patch size, a novel MEF framework as shown in Figure 2 is proposed. First, texture-cartoon decomposition is applied to obtain image texture components. Then, image texture entropy is calculated to achieve the adaptive selection of image patch size. Third, the structural patch decomposition approach is utilized to obtain the initial fused image. Finally, the color MEF structural similarity index is used to iteratively optimize the initial fused image to get the final fused image.

### 2.1. Image Cartoon-Texture Decomposition

**Figure 3:**
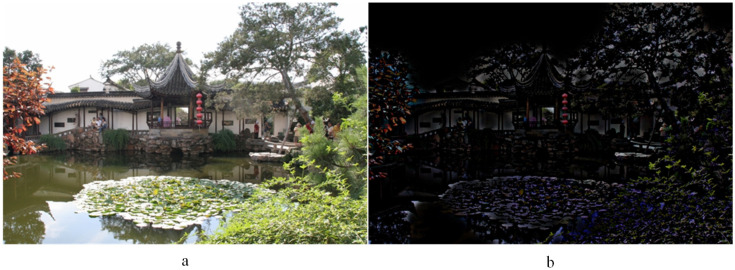
(**a**) The source image; (**b**) the texture component.

The texture and cartoon components of the image describe the detailed and structured information, respectively [31,32]. In the proposed fusion framework, texture and cartoon components are obtained by decomposing input images. In this work, image texture decomposition is achieved by implementing the regularization Vese-Osher (VO) model [33,34]. The VO model is shown as Equation (1):

(1) $$\begin{array}{c} \begin{array}{c} \underset{u,\vec{g}}{\inf}\left\{VO_{P}\left(u,\vec{g}\right)={\left|u\right|}_{TV}+\lambda {∥f-udiv(\vec{g})∥}_{L^{2}}^{2}+\mu {∥\vec{g}∥}_{L^{P}}\right\} \end{array}\;\; \end{array}$$

where ${∥\vec{g}∥}_{L^{P}}={\left[\int {\left(\sqrt{g_{1}^{2}+g_{2}^{2}}\right)}^{P}dxdy\right]}^{\frac{1}{P}}$ represents the $L^{P}$ norm of $\vec{g}$, the value of *p* being between one and 10. The $g=\left(g_{1},g_{2}\right)$ is a vector to represent digital images in *G* space, and λ and μ are regularization parameters. In Equation (1, the first term *u* is the cartoon component of the image. The second term $f-u-div\left(\vec{g}\right)$ together with the third term $∥\vec{g}∥$ ensures that $v=f-u\approx div\left(\vec{g}\right)$, which represents the image minus the rest of the cartoon. *v* is the texture component of the image. When λ→∞ and P→∞, then in the limit, $f-u=div\left(\vec{g}\right)$ almost everywhere for those $\vec{g}$. Therefore, in the limit, the middle part in Equation (1) will disappear, and the third part becomes ∥f−u∥, which represents the texture component of the image. *f* represents the input image. The cartoon component of image *u* can be obtained by the Euler–Lagrange equation, shown in Equation (2): Once the cartoon component is obtained, the texture component can be simply calculated using v=f−u. Figure 3a represents the source image, and Figure 3b is the texture component obtained by the VO model. For more information, please refer to [34].

(2) $$\begin{array}{c} \begin{array}{c} u=f-\partial_{x}g_{1}-\partial_{y}g_{2}+\frac{1}{2\lambda }div\left(\frac{\nabla u}{\left|\nabla u\right|}\right) \end{array}\;\; \end{array}$$

### 2.2. Adaptive Selection of Image Patch Size

**Figure 4:**
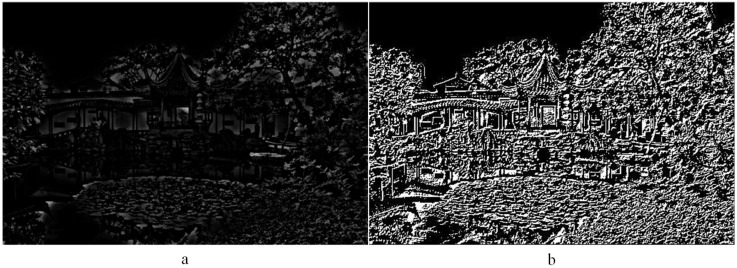
(**a**) The texture image in gray space; (**b**) the differential image.

The proposed adaptive selection of image patch size (APS) algorithm applies the statistical method to the grayscale difference to calculate the entropy value of image texture features. The details of the proposed APS algorithm are shown as the following steps:

Step 1: This converts image texture components into a grayscale image. The grayscale image is shown in Figure 4a. (x,y) denotes a point in the image. A point that has quite a small distance from (x,y) is denoted as $\left(x+\Delta x,y+\Delta y\right)$. Its grayscale difference value can be represented as Equation (3). Then, a grayscale differential image is obtained.

(3) $$\begin{array}{c} \begin{array}{c} g_{\Delta }\left(x,y\right)=g\left(x,y\right)-g\left(x+\Delta x,y+\Delta y\right) \end{array}\;\; \end{array}$$

where $g_{\Delta }$ denotes the gray-value difference. Letting (*x*, *y*) move over the entire image, then a grayscale differential image is obtained. Figure 4b is a grayscale differential image obtained by the gray difference algorithm.

Step 2: Assuming that all possible values of the grayscale difference have *m* levels, it calculates the entropy value of the image texture features. A histogram of $g_{\Delta }$ is obtained by letting (x,y) move over the entire image and counting the number of times for each value of $g_{\Delta }$. $p\left(i\right)$ is the probability value of each gray-level difference obtained from histogram statistics. The entropy value of image texture is obtained by Equation (4).

(4) $$\begin{array}{c} \begin{array}{c} ent=-\sum_{i=0}^{m}p\left(i\right)\log_{2}\left[p\left(i\right)\right] \end{array}\;\; \end{array}$$

Step 3: After iterating the above processes for all input images, this algorithm obtains the entropies of all image texture features as $\left\{ent_{1},ent_{2},\ldots ,ent_{n}\right\}$, where *n* represents the number of input images.

**Figure 5:**
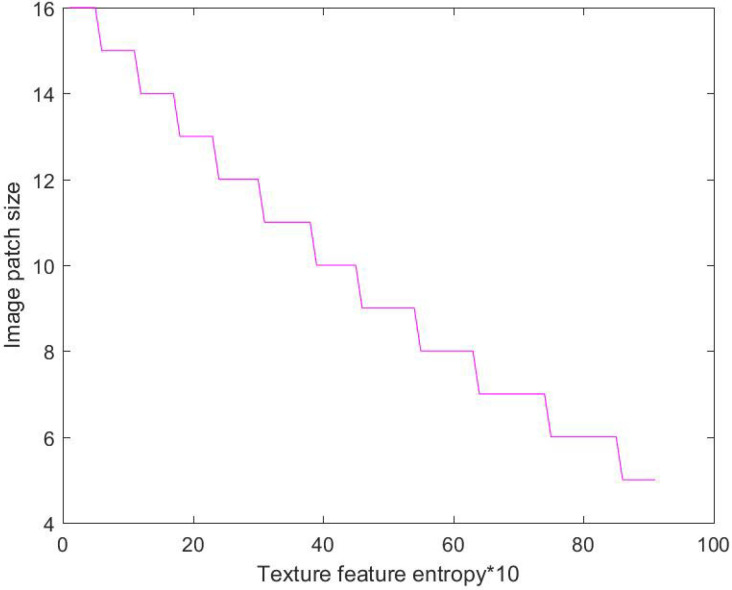
Relationship between image texture entropy and image patch size.

Step 4: Adaptive selection of the image patch size is obtained, according to the entropy value of the image texture feature. Based on the gray-level co-occurrence matrix, the obtained texture entropy value can reflect the coarseness of image texture to a large extent. When the entropy value becomes smaller, the texture is finer. On the contrary, when the entropy value becomes bigger, the texture is coarser [35]. The optimal image patch size is closely related to the coarseness of image texture. When image texture becomes coarser, a larger image patch size should be selected in image decomposition to ensure the good performance of the texture structure in decomposed components. Conversely, when image texture gets finer, a smaller image patch size should be selected to achieve a better texture synthesis effect. In this paper, the coarseness of image texture is characterized according to the entropy value of image texture, and the optimal image patch size is automatically selected. When the image texture entropy value is small, this indicates that the texture is fine, and a larger image patch size should be selected. When the image texture entropy value is larger, this indicates that the texture is rough, and a smaller image patch size should be selected [36]. For the 24 sets of multi-exposure source image sequences used in the experiment, a 16 size image patch size was selected for image fusion. For each set of texture images, the image patch size is adjusted from large to small, and the fusion result is compared to find a reasonable parameter range of the image patch size, as shown in Figure 5. The abscissa represents the entropy of different texture images, arranged from small to large, and the ordinate represents the optimal image patch parameters of the corresponding texture image. It can be seen from Figure 5 that as the image texture entropy value increases, the optimal image patch size parameter decreases, and the hyperbolic function can be used for fitting. The empirical formula for the optimal image patch size is shown as Equation (5).

(5) wSize=pSize×0.1×ENTENT1010ENT−ENTENT1010−ENTENTENT1010ENT+ENTENT1010−ENT+pSize×e−ENT

where ENT is the mean of image texture entropy values, $ENT=\frac{1}{n}\sum_{i=1}^{n}ent_{i}$, pSize is the preset image patch decomposition size, and pSize=21. Thus, the corresponding optimal matching patch size is wSize×wSize.

### 2.3. Structure Patch Decomposition and Structural Similarity Optimization for MEF

The image patches obtained by the APS algorithm are decomposed into three components to obtain an initial fused image by using the structure patch decomposition algorithm [3,37]. Then, the initial fused image is optimized by the MEF-SSIMc algorithm [29] to obtain the final fused image. Specifically, the algorithm details are shown as follows:

Step 1: Patches of the same spatial position are extracted from the image sequence processed by the APS algorithm using a dynamic stride *D*.

Step 2: Three conceptually-irrelevant components, signal strength $c_{i}$, signal structure $s_{i}$, and mean intensity $l_{i}$, are obtained by applying the structure patch decomposition approach to an image patch.

Step 3: Three components are processed separately. The signal strength component is processed first. As shown in Equation (6), the maximum signal strength of all source image patches in the same spatial location is selected as the signal strength of the fused image patch. The local contrast determines the texture and structure visibility of the local image patch. Generally, when local contrast becomes higher, the visibility of the local image patch is better. In this paper, local contrast is directly related to signal strength.

(6) $$\begin{array}{c} \begin{array}{c} \hat{c}=\max_{1\leq i\leq n}c_{i}=\max_{1\leq i\leq n}∥{\tilde{x}}_{i}∥ \end{array}\;\; \end{array}$$

where ∥·∥ represents the $l_{2}$ and ${\tilde{x}}_{i}$ is local contrast.

For signal structure $s_{i}$, in order to make the fused image patch represent the structures of all source image patches, a weighted average processing signal structure as shown in Equation (7) is applied.

(7) s^=∑inSx˜isi∑inSx˜isi∑inSx˜i∑inSx˜i∥∑inSx˜isi∑inSx˜isi∑inSx˜i∑inSx˜i∥

where $S\left(\cdot \right)$ is a weighting function defined as $S\left({\tilde{x}}_{i}\right)={∥{\tilde{x}}_{i}∥}^{4}$. Similar to Equation (7), a weighted average process as shown in Equation (8) is applied to mean intensity $l_{i}$.

(8) $$\begin{array}{c} \begin{array}{c} \hat{l}=\frac{\sum_{i=1}^{n}L\left(\mu_{i},l_{i}\right)l_{i}}{\sum_{i=1}^{n}L\left(\mu_{i},l_{i}\right)} \end{array}\;\; \end{array}$$

where $L\left(\cdot \right)$ defines a weighting function. It quantifies the well-exposedness of the local image patch in the source image and performs the calculation using the Gaussian model.

(9) $$\begin{array}{c} \begin{array}{c} L\left(\mu_{i},l_{i}\right)=\exp\left(-{\left(\frac{\mu_{i}-0.5}{2\sigma_{g}^{2}}\right)}^{2}-{\left(\frac{l_{i}-0.5}{2\sigma_{l}^{2}}\right)}^{2}\right) \end{array}\;\; \end{array}$$

where $\mu_{i}$ and $l_{i}$ denote the global mean intensity of the source image and the local mean intensity of current patch, respectively, and $\sigma_{g}$ and $\sigma_{l}$ are the Gaussian standard deviation. In this paper, $\sigma_{g}$ and $\sigma_{l}$ are 0.2 and 0.5, respectively.

Step 4: When $\hat{c}$, $\hat{s}$, and $\hat{l}$ are calculated, a new uniquely fused image patch can be obtained by recombination.

(10) $$\begin{array}{c} \begin{array}{c} \hat{x}=\hat{c}\cdot \hat{s}+\hat{l} \end{array}\;\; \end{array}$$

Step 5: In this fused framework, all source image sequences iterate Processes 1–4 above to obtain all fused patches. Then, the fused patches are aggregated to achieve the initial fused image.

Step 6: In this step, the MEF-SSIMc algorithm [29] defines the structural similarity index (SSIM) to evaluate the image patch quality.

(11) $$\begin{array}{c} \begin{array}{c} S\left(\left\{x_{i}\right\},y\right)=\frac{\left(2\mu_{\hat{x}}\mu_{y}+C_{1}\right)\left(2\sigma_{\hat{x}y}+C_{2}\right)}{\left(\mu_{\hat{x}}^{2}+\mu_{y}^{2}+C_{1}\right)\left(\sigma_{\hat{x}}^{2}+\sigma_{y}^{2}+C_{2}\right)} \end{array}\;\; \end{array}$$

where $\left\{x_{i}\right\}=\left\{x_{i}|1\leq i\leq n\right\}$ represents the group of image patches at the same location in the sequence of source images, $\mu_{\hat{x}}$ and $\mu_{y}$ represent the average intensity of fused image patch $\hat{x}$ and the referenced image patch *y*, respectively, $\sigma_{\hat{x}}^{2}$ and $\sigma_{y}^{2}$ represent the local deviation of $\hat{x}$ and *y* respectively, $\sigma_{\hat{x}y}$ is the local covariance between $\hat{x}$ and *y*, and $C_{1}$ and $C_{2}$ are small constants that satisfy $C_{1}>0,C_{2}>0$ to avoid the algorithm becoming unstable when the denominator approaches zero.

Step 7: As shown in Equation (12), the fused image overall quality score is obtained by averaging the SSIM index of fused image patches.

(12) $$\begin{array}{c} \begin{array}{c} Q\left(\left\{x_{i}\right\},Y\right)=\frac{1}{N}\sum_{j=1}^{N}S\left(\left\{R_{j}X_{i}\right\},R_{j}Y\right) \end{array}\;\; \end{array}$$

where *N* represents the number of image patches, $R_{j}$ denotes a binary matrix, the number of its columns is equal to the image dimension, the number of rows is equal to the patch size ${CN}^{2}$, *C* is the number of color channels, and *N* represents the patch size.

Step 8: The SSIM index is updated by using gradient iterations. As illustrated in Equation (13), the SSIM index $Y_{i}$ obtained by the *i*-th iteration is improved by using the gradient ascent algorithm to achieve image optimization.

(13) $$\begin{array}{c} \begin{array}{c} Y_{i+1}=Y_{i}+\lambda \nabla_{Y}Q\left(\left\{X_{i}\right\},Y\right){|}_{Y=Y_{i}} \end{array}\;\; \end{array}$$

where $\nabla_{Y}Q\left(\left\{X_{i}\right\},Y\right)$ denotes the gradient of $Q\left(\left\{X_{i}\right\},Y\right)$, and the details of calculating $\nabla_{Y}Q\left(\left\{X_{i}\right\},Y\right)$ are described in [29]. λ denotes a step parameter controlling the speed of movement in the image. When $\left|Q_{i+1}-Q_{i}\right|<\varepsilon =10^{-6}$ is satisfied, the iteration stops, and the final fused image is obtained.

## 3. Experiments and Analyses

### 3.1. Experiment Preparation

In this section, 24 sets of multi-exposure source image sequences that describe diverse scenes containing different shades regions with disparate colors were used in comparative experiments. All the source image sequences were collected by Ma [29] and can be downloaded form https://ece.uwaterloo.ca/~k29ma/. Ten different MEF methods, such as Bruce13 [12], Gu12 [23], Mertens07 [10], Shen14 [14], Ma17 [3], SSIM-MEF [3,29], Proposed-8, Proposed-16, Proposed-24, and the proposed APS-MEF solution were applied to 24 sets of multi-exposure source image sequences for comparison. All of the fused images were either collected by Ma [29] or generated by open source codes. All the experiments were programmed in MATLAB 2016a (MathWorks, Natick, MA, USA) on an Intel^®^ Core^TM^ i7-7700k CPU @ 4.20-GHz desktop with 16.00 GB RAM.

#### Objective Evaluation Metrics

To evaluate quantitatively the quality of a fused image, a single evaluation metric cannot fully reflect the quality of the fused image. Therefore, several metrics were applied to make as comprehensive an evaluation as necessary. In these experiments, three objective evaluation indexes, $Q^{AB/F}$ [38,39], MI [40,41], and $Q^{CB}$ [42,43], were selected to quantify the fused results of different MEF methods.

As a gradient-based quality index, $Q^{AB/F}$ [38,39] and MI [40,41] were used to measure the edge information and the similarity between the fused image and the source images, respectively. $Q^{CB}$ [42,43], as a human perception-inspired fusion metric, was used to evaluate the human visualization performance of the fused image.

### 3.2. Experiment Results and Analyses

#### Experiment Results of Six MEF Methods

We conducted the following comparative experiments to prove that the proposed APS-MEF algorithm can achieve excellent fusion performance in human visual observation. Twenty four sets of multi-exposure source images were fused by ten MEF methods, which were Bruce13 [12], Gu12 [23], Mertens07 [10], Shen14 [14], Ma17 [3], SSIM-MEF [3,29], Proposed-8, Proposed-16, Proposed-24, and the proposed APS-MEF. The SSIM-MEF method is the result of the optimization of Ma17 [3] using SSIM-MEF [29]. Proposed-8, Proposed-16, and Proposed-24 represent the fusion results of the proposed algorithm using 8×8, 16×16,and 24×24 fixed image patch sizes, respectively. The selected patch sizes of the 24 sets of multi-exposure images in the proposed APS-MEF algorithm are shown in Table 1. In total, 240 fused images were obtained and divided into 24 groups according to the scene content. Five sets were selected from the total of 24 sets of fused images for demonstration in this paper.

**Figure 6:**
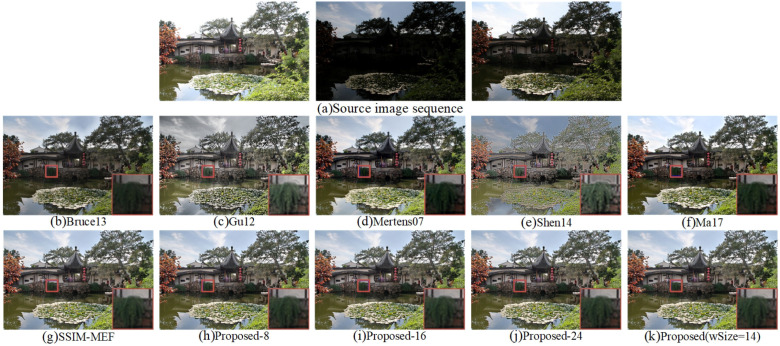
Visual comparison of the fused “Chinese Garden” between the proposed method and nine existing methods.

All fused images of “Chinese Garden” obtained by ten different methods are illustrated in Figure 6. Compared with Bruce13 [12], the fused image obtained by the proposed ASP-MEF contained more structure and texture details in the pool and corridor areas, and it achieved excellent performance in global contrast. Moreover, the fused image obtained by the proposed method had more vivid color and comfortable visual effects than the fused ones of Gu12 [23] and Shen14 [14]. The color of the fused image obtained by Gu12 [23] was distorted; for example, the sky is gray in Figure 6c. The fused image produced by Shen14 [14] had sharp intensity changes and unnatural colors that were either saturated or pale. According to the details of the fused images shown in Figure 6, the plants shown in the fused images by Bruce13 [12] and Ma17 [3] had unclear structure and texture details. Although images fused by Gu12 [23] and Shen14 [14] had good local details, the global visual effects were poor. The fused image obtained by the SSIM-MEF [3,29] method had high saturation. Compared with other MEF methods, the proposed APS-MEF method not only ensured the articulation of local details, but also achieved contrast and color saturation that conformed to the human visual observation. In addition, compared to the Proposed-8, Proposed-16, and Proposed-24 fusion methods, our proposed ASP-MEF performed better with respect to the human visual system.

**Figure 7:**
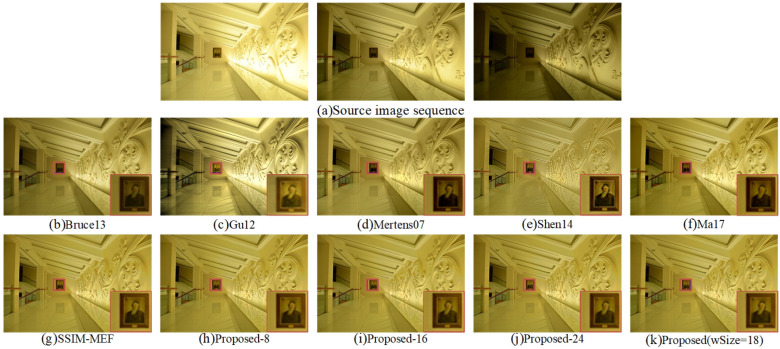
Visual comparison of the fused “Yellow Hall” between the proposed method and nine existing methods.

The fused “Yellow Hall” images by the ten methods are shown in Figure 7. The fused image obtained by Bruce13 [12] had poor overall brightness, and the details of dark regions shown in the source images could not be well represented. Although Figure 7c,d shows good performance in global contrast, both of them had distortions to varying degrees. The color saturation of stair areas shown in the fused result of Gu12 [23] was poor. Due to the high sharpness of the fused image by Shen14 [14], the edges of wall were unsmooth. The color appearances of the wall and stair areas in the fused images by Mertens07 [10], Ma17 [3], and APS-MEF were relatively natural and consistent with the source images. However, the edge details of portraits shown in the local enlarged areas of Figure 7d,f were blurred, and the brightness was dark. The color saturation of the wall relief of the Proposed-24 method fusion result was slightly worse than that of the proposed APS-MEF, SSIM-MEF [3,29], Proposed-8, and Proposed-16 methods. Compared to the proposed APS-MEF and SSIM-MEF [3,29] methods, the wall relief edge of the Proposed-8 and Proposed-16 fusion results was smoother. Although the proposed APS-MEF and SSIM-MEF [3,29] method performed excellent in the color and local detail of the image, the overall appearance of the proposed APS-MEF method fusion result was brighter. Therefore, compared to the other nine fused results, the image fused by the proposed APS-MEF method had clearer local texture details, as well as a brighter and warmer overall appearance.

**Figure 8:**
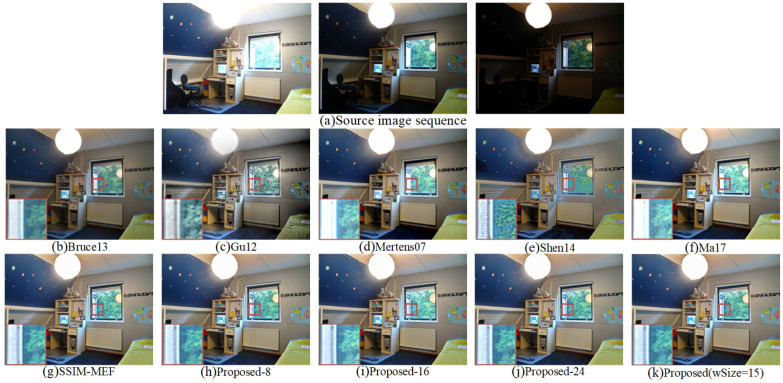
Visual comparison of the fused “Window” between the proposed method and nine existing methods.

The fused “Window” images are demonstrated in Figure 8. In the fused image obtained by Bruce13 [12], the light brightness was weak, and the local area details of the window were blurred. Compared to the fused images of Shen14 [14] and APS-MEF, there were obviously black shadows from the lights in Figure 8e and a black shadow on the wall. Besides, the structure and texture of the scene outside the window were not obvious. In the fusion result of Gu12 [23], the colors of the bed, wall, and portrait were obviously distorted. The chair shown in Figure 8d had a weak brightness and blurry edge. The local enlargement of the fused image obtained by the Ma17 [3] method had a high exposure, and the detail information was blurred. Compared to the Proposed-8 and Proposed-16 methods, the proposed method was moderately bright. The fused images of the proposed method, SSIM-MEF [3,29], and Proposed-24 were natural in color and brightness. The proposed method had much clearer edge and structure details of the scene outside the window. Therefore, compared with the other nine methods, the fused image obtained by the proposed method was more natural with respect to human vision and had better local details.

**Figure 9:**
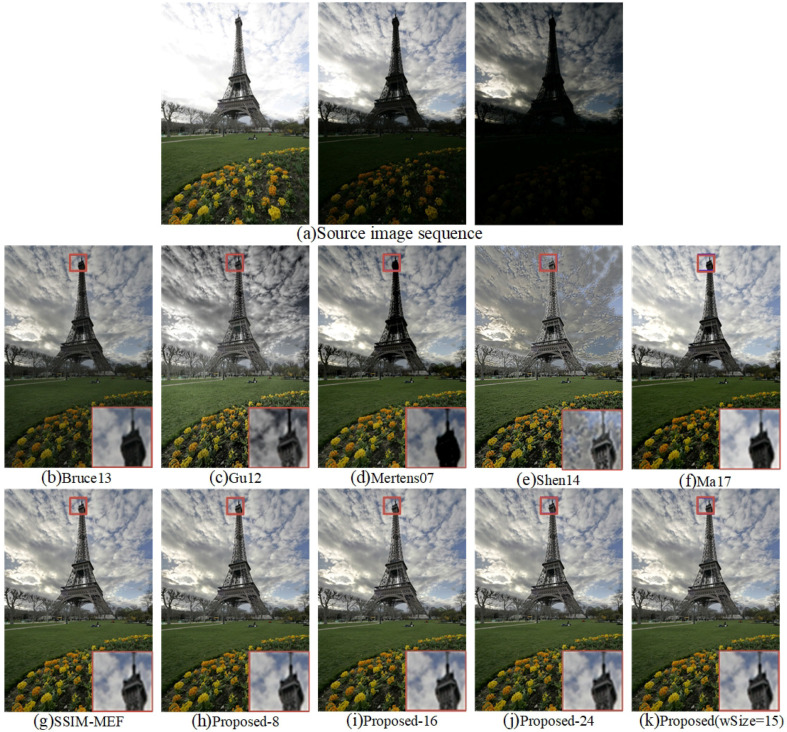
Visual comparison of the fused “Tower” between the proposed method and nine existing methods.

Figure 9 shows ten fused images of “Tower” generated by the ten different methods. Towers in the fusion results of Bruce13 [12] and Mertens07 [10] were black and indistinct. The magnified local images clearly show that the interior details of the tower were missing. Although the details of the tower in Figure 9d are clear, the brightness of the clouds is overexposed, as well as the colors of the clouds and sky are obviously distorted. The edge of the cloud in the fused image of Shen14 [14] was too sharp. Moreover, the overall color was not soft, and the visual effect was poor. The fused image obtained by Ma17 [3] had a high exposure for the the clouds, which weakened the detailed texture information. The fused images obtained by the Proposed-16 and Proposed-24 methods were similar to human visual perception. SSIM-MEF [3,29], Proposed-8, and the proposed method reached the best overall visual effect. It was difficult to distinguish the difference between the Proposed-16, Proposed-24, SSIM-MEF [3,29], Proposed-8, and the proposed APS-MEF fusion results by human visual observation.

**Figure 10:**
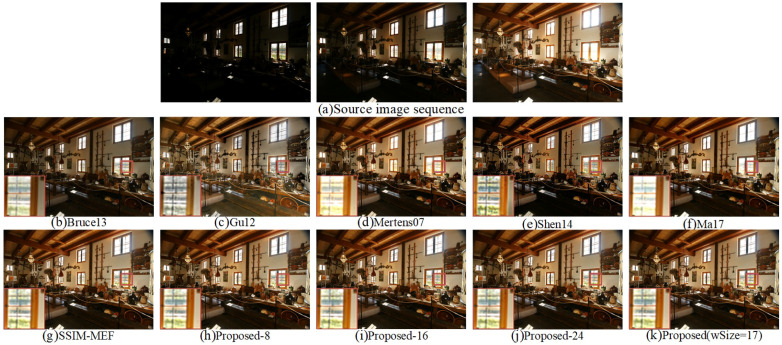
Visual comparison of the fused “Farmhouse” between the proposed method and nine existing methods.

The ten fused images of “Farmhouse” are shown in Figure 10. As shown in Figure 10b, the overall brightness of Bruce 13 [12] is weak, and the details of some dark areas cannot be well demonstrated. The overall color and brightness of Gu12 [23] was natural, but the color of the marked area outside the window was obviously distorted. The brightness of the bottom part shown in the fused images obtained by Mertens07 [10] and Shen14 [14] was dark. At the same time, the colors of the small ornaments were not natural. Compared with Ma17 [3], the fused image of the proposed method presented more details outside the window. Compared with the SSIM-MEF [3,29] and Proposed-24 methods, the proposed method had moderate brightness. The fused images obtained by the Proposed-8 and Proposed-16 methods performed similarly to the human visual system. The fused image obtained by the proposed method had the best overall performance in brightness, local detail processing, and visual effect among all ten image fusion methods.

### 3.3. Objective Evaluation Metrics

**Figure 11:**
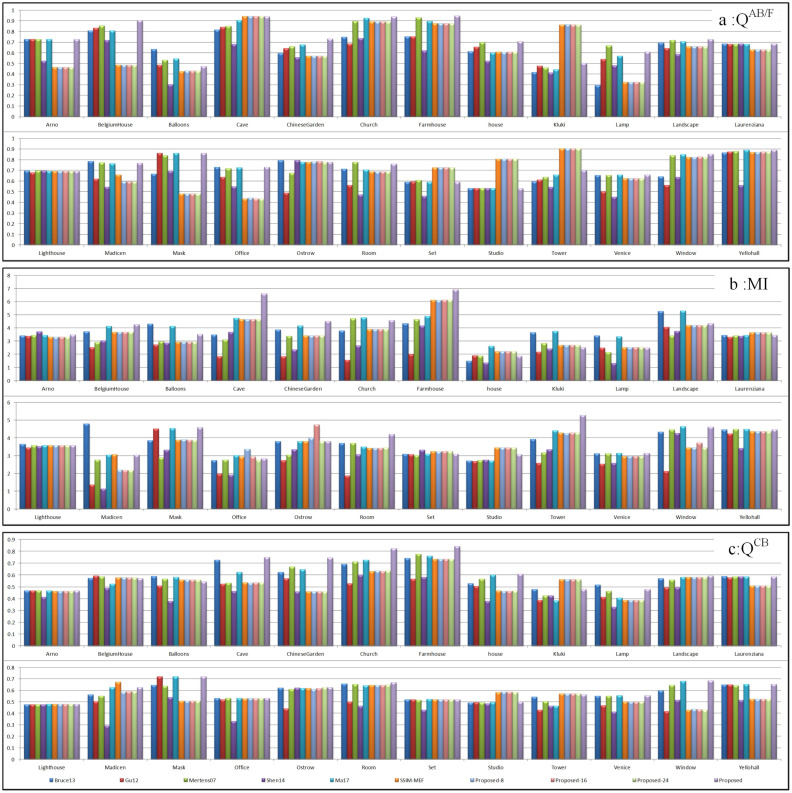
Objective evaluations of 24 source image sets in the MEF experimentation.

In this paper, four objective evaluation indicators were used to evaluate the fusion performance objectively from four aspects: edge preservation, similarity, human visual effects, and calculation time. The average values of the 24 sets for the comparative experiments obtained by ten different methods in three objective evaluation indexes are shown in Table 1. The objective evaluation results of the 24 sets of multi-exposure fusion images are shown in Figure 11. In Figure 11, the three bar charts represent $Q^{AB/F}$, MI and $Q^{CB}$ objective values, respectively. As can be seen from Figure 11, in all of the comparison methods, our method ranks as the top two in most cases for the three objective indicators and all 24 groups of multi-exposure image fusion problems. Concretely, the $Q^{AB/F}$ index scores of the proposed fusion results were ranked as top three in 20 groups. There were 16 groups in which the MI scores of the proposed fusion results were ranked in the top three place. For the $Q^{CB}$-index score, the proposed method had the top three highest scores in 22 groups. From Figure 11, among the three objective evaluation indicators of all fusion results, the objective evaluation scores of the proposed method were in the top three for 80% of the results, and the remaining 20% of the results of the fusion objective scores were in the middle. This indicated that the proposed method could better preserve the details of the source scene and obtain better human visual effects.

From Table 2, it is indicated that the fused image obtained by Shen14 [14] had the lowest value for $Q^{AB/F}$ and $Q^{CB}$. Except Gu12 [23], the MI value of Shen14 [14] was also lower than the other eight fusion methods. This means that the fusion result of Shen14 [14] did not have good performance in image edge processing and human visual effects. In Figure 6, Figure 7 and Figure 8, the fused images of Shen14 [14] have excessive sharpening, which leads to a poor visual effect and poor edge detail processing of the local magnified region. The objective and subjective evaluations of Shen14 [14] were almost the same. Compared with the other nine fusion methods, the MI value of Gu12 [23] was the lowest, which indicates that the similarity between the fused image and source images was objectively the worst. According to the previous subjective comparison, the fused images of Gu12 [23] shown in Figure 6, Figure 8 and Figure 9 had a color distortion issue. The objective evaluation values indicate the same result as the subjective comparison. The values of SSIM-MEF [3,29] in the chart were the result of the optimization of Ma17 [3] using SSIM-MEF. It can be observed that the $Q^{AB/F}$ and $Q^{CB}$ evaluation scores of the optimized image were higher than the unoptimized image, which implies that the quality of the image of Ma17 [3] was good after SSIM-MEF [3,29] optimization. As can be seen in Figure 6, the color of the fusion image of SSIM-MEF was more natural than that of Ma17 [3], and the edge detail of the tree were clearer. The same conclusion can be drawn from both subjective and objective evaluation. Proposed-8, Proposed-16, and Proposed-24 differed with the proposed method only in the size selection of image patches, but the objective indices of fused images were completely different. Their values were lower than those of the proposed method and better than those of a few other comparative experiments. It can be inferred that the method of adaptive structure selection was superior to the method of fixed structure. The proposed ASP-MEF obtained the maximum values in all three indexes. This confirms that the proposed method had good performance in edge detail processing and visual effect, and the fused image of proposed method had high similarity to the source images. From Table 2, the calculation time of Shen14 [14] was the longest, Proposed-24 the second, and the time difference of other methods was not obvious. Therefore, except for the Shen14 [14] method, the difference in fusion complexity and efficiency of the remaining algorithms was not significant. However, the proposed method performed better than the other methods in the other three objective evaluation indicators. In addition, compared with the other nine methods, subjectively, the proposed method also achieved optimal performance in color softness, brightness, and local detail processing. In conclusion, the proposed method was the best in terms of both subjective comparison and objective evaluation.

## 4. Conclusions

This paper proposes a novel MEF method named the adaptive patch structure-based MEF (APS-MEF). First, texture-cartoon decomposition is applied to obtain image texture components. Second, the image texture entropy is calculated to achieve the adaptive selection of image patch size. Then, the structural patch decomposition approach is utilized to obtain the initial fused image. Finally, the initial fused image is iteratively optimized by applying the color MEF structural similarity index to obtain the final fused image. The proposed algorithm evaluates the local information by texture entropy and adaptively selects image patch size, which allows the fused image to contain more detailed information. The visual quality of the fused image is improved by combining the decomposition of the image patch structure and the similarity index algorithm of the color image structure. The comparative experiments show that the proposed APS-MEF fusion method can preserve more detailed information and obtain better human visual effects.

The proposed APS-MEF uses SSIMc-MEF as the iterative optimization algorithm. However, the iterative optimization algorithm is not suitable for real-time applications. In the future, an efficient non-iterative optimization algorithm will be adopted to improve the efficiency of the fusion algorithm.

## Figures and Tables

**Table 1:** The selected patch sizes of the 24 sets of multi-exposure images.

Image Set	Patch Size	Image Set	Patch Size
Arno	13	Balloons	15
BelgiumHouse	8	Cave	10
Chinese Garden	14	Church	9
Farmhouse	17	House	10
Kluki	12	Lamp	13
Landscape	12	Laurenziana	12
Lighthouse	12	MadisonCapitol	9
Mask	10	Office	17
Ostrow	18	Room	15
Set	13	Studio	12
Tower	15	Venice	10
Window	15	Yello wHall	18

**Table 2:** Objective evaluation of the ten MEF methods.

	*Q* ^*AB*/*F*^	MI	*Q^CB^*	Time
Bruce13	0.66684	3.67199	0.57956	17.30 s
Gu12	0.64301	2.61998	0.50975	13.60 s
Mertens07	0.71941	3.26387	0.57021	10.20 s
Shen14	0.57109	2.93935	0.46300	57.27 s
Ma17	0.71470	3.85767	0.57580	13.64 s
SSIM-MEF	0.72586	3.67061	0.57730	15.93 s
Proposed-8	0.65852	3.50575	0.53225	**9.50 s**
Proposed-16	0.65863	3.53180	0.53234	14.11 s
Proposed-24	0.65814	3.47528	0.53253	21.13 s
Proposed	**0.73623**	**3.91869**	**0.60737**	14.12 s
